# Comparing Pilates and Gym Ball Exercises for Primary Dysmenorrhea Management: An Empirical Study

**DOI:** 10.7759/cureus.59184

**Published:** 2024-04-28

**Authors:** Veena Kirthika S, Sudhakar S, Mohan Kumar G, Ramachandran S, Deepthi RNV, Senthil Selvam P

**Affiliations:** 1 Physiotherapy (Neurology), Faculty of Physiotherapy, Dr. M.G.R. Educational and Research Institute (Deemed to be University) Maduravoyal, Chennai, IND; 2 Physiotherapy (Sports), Faculty of Physiotherapy, Dr. M.G.R. Educational and Research Institute (Deemed to be University) Maduravoyal, Chennai, IND; 3 Physiotherapy (Orthopedics), Faculty of Physiotherapy, Dr. M.G.R. Educational and Research Institute (Deemed to be University) Maduravoyal, Chennai, IND; 4 Physiotherapy (Cardiovascular and Pulmonary), Faculty of Physiotherapy, Dr. M.G.R. Educational and Research Institute (Deemed to be University) Maduravoyal, Chennai, IND; 5 Physiotherapy(Obstetrics and Gynecology), Faculty of Physiotherapy, Dr. M.G.R. Educational and Research Institute (Deemed to be University) Maduravoyal, Chennai, IND; 6 Physiotherapy, School of Physiotherapy, Vels Institute of Science, Technology and Advanced Studies, Chennai, IND

**Keywords:** menstrual distress questionnaire, visual analog scale, swiss ball exercise, pilates exercise, menstruation, primary dysmenorrhea

## Abstract

Background: Primary dysmenorrhea, commonly known as menstrual cramps, is a prevalent gynecological issue that impacts many women in their childbearing age. It manifests as reoccurring, cramp-like lower abdominal pain, usually commencing right prior to or during the menstrual period. These painful sensations can be severe, extending to the lower back and upper thighs can greatly disrupt a woman's daily life and overall well-being. The optimal exercise approach is needed for individuals seeking relief from primary dysmenorrhea, allowing healthcare providers and women themselves to make informed decisions regarding their treatment options. Though many forms of exercise interventions exist in the treatment of primary dysmenorrhea, this study aims to compare two forms of intervention namely pilates and gym ball exercises on pain and menstrual distress among women with Primary Dysmenorrhea.

Methods: This experimental comparative study was carried out with 30 young female participants over a 12-week period. Participant recruitment was done through a simple random sampling method. The criteria of inclusion encompassed young females aged 17-25 years, those in good health, and those experiencing primary dysmenorrhea. Exclusion criteria included secondary dysmenorrhea, medication use, polycystic ovarian syndrome, bleeding disorders, positive pregnancy tests, breastfeeding, and other medical conditions. Group A received Pilates exercises, while Group B underwent Gym ball exercises. The assessment of both groups' menstrual distress levels was conducted using the Moos Menstrual Distress Questionnaire (MDQ) and pain scores using the Visual Analog Scale (VAS).

Result: In the statistical analysis, Group A (Pilates Exercises) showed a significantly lower mean value (2.60) on the VAS compared to Group B (Swiss Ball Exercises) (5.46), with both groups experiencing a notable reduction in post-test scores (p ≤ 0.001). Additionally, on the MOOS MDQ score, Group A (Pilates Exercises) achieved a lower mean value (79.33) compared to Group B (Swiss Ball Exercises) (103.26), with both groups demonstrating a significant decrease in post-test scores (p ≤ 0.001).

Conclusion: In conclusion, this study underscores the significance of exercise therapy, particularly Pilates exercises, as a holistic approach to addressing primary dysmenorrhea, improving physical well-being, and enhancing the overall quality of life.

## Introduction

Primary dysmenorrhea, often referred to as myalgia, is identified by lower region abdominal pain that can extend to the thighs and the upper part of the spine. It is frequently combined with symptoms like nausea, headaches, fatigue, and also diarrhea [1]. Dysmenorrhea is defined as a painful experience during menstruation. It is a widespread issue in India, impacting a substantial portion of young females. Research indicates that an estimated 60% to 90% of menstruating women in India encounter dysmenorrhea to varying degrees. This condition is most frequently observed among adolescent and young adult women, often commencing shortly after the initiation of menstruation. Its prevalence is particularly high among females aged 15 to 30 years [2]. It significantly hampers daily activities and is also the predominant reason for short-term absence from school and work disruptions among women under the age of 30 [3].

Dysmenorrhea is categorized into two main types: primary and secondary, depending on the presence or absence of any underlying causes. Secondary dysmenorrhea typically is associated with any underlying pathologies of the pelvis. Primary dysmenorrhea, which is painful periods with menstrual cramps, is characterized by painful occurrences during the menstrual cycle [4,5]. Typically, this pain starts a day prior to or during the first day of menstruation and subsides during the end of the menstrual period [6].

The pain experienced in primary dysmenorrhea is appertaining to excessive prostaglandins (PGs) production during the menstrual cycle, which are chemical substances present in the uterine lining contributing to cramps and pain. This overproduction of PGs is hypothesized to lead to increased uterine contractions, resulting in insufficient oxygen supply and reduced blood flow to the uterus, which ultimately causes the pain and cramping associated with primary dysmenorrhea. Factors that increase the likelihood of experiencing primary dysmenorrhea include an earlier onset of menstruation and a significant volume of menstrual bleeding, smoking, a positive family history of the condition, obesity, and alcohol consumption [7].

Managing primary dysmenorrhea is a critical concern for both healthcare providers and women themselves, as the pain and associated symptoms can be debilitating. While various pharmacological treatments are available, which mainly include NSAIDs, their side effects like headache, indigestion as well as drowsiness are a major concern. Hence there is a growing interest in non-pharmacological approaches, particularly exercise-based interventions, as they offer potential relief without the side effects associated with medications.

Among the exercise modalities gaining attention for primary dysmenorrhea management, two stand out prominently: Pilates and gym ball exercises. Pilates, a mind-body exercise system that emphasizes core strength, flexibility, and also body awareness, has been increasingly utilized as a holistic approach to improving overall physical health and well-being [8]. On the other hand, gym ball exercises focus on strengthening muscles in the abdominal, leg, arm, and back regions. Additionally, they improve balance, stability, and blood circulation, ultimately contributing to the alleviation of menstrual pain [9]. Intense physical activity is thought to trigger the secretion of beta-endorphins, which function like natural painkillers, thereby alleviating menstrual pain often connected with primary dysmenorrhea [10].

Despite the rising interest in exercise-based interventions for primary dysmenorrhea, there remains a gap in the understanding of which specific exercises, such as Pilates or gym ball exercises, could be more effective in alleviating symptoms in this condition. This empirical study aims to bridge this gap by conducting a comprehensive investigation into the comparative effectiveness of Pilates and Gym ball exercises as non-pharmacological interventions on pain and menstrual distress experienced by subjects with primary dysmenorrhea [11,12].

## Materials and methods

This experimental comparative study was conducted at the Faculty of Physiotherapy, DR M.G.R Educational and Research Institute, Chennai, Tamil Nadu, India from February 2023 to May 2023 involving a sample of 30 young females. The priori sample size has been estimated based on the anticipated effect size of at least, d≥0.8 using G * Power 3.1.9.4 software [13]. This study spanned a duration of 12 weeks, and the subjects were chosen through a random sampling method Inclusion criterion encompassed young females aged 17-25 years who were ambulatory and generally healthy but suffered from primary dysmenorrhea [14]. Exclusion criteria included individuals with secondary dysmenorrhea, those on medication, diagnosed with polycystic ovarian syndrome, bleeding disorders, positive pregnancy tests, breastfeeding, medical disorders, or primary dysmenorrhea attributed to other causes.

The study's need was thoroughly elaborated to all participants, and their informed consent was obtained. The study received ethical clearance from University Research and Ethics Committee (ACSMCH/Ethical(42)/02-2023) as well as adhered meticulously to guidelines mentioned in the 2013 Helsinki Declaration, which is endorsed by the World Medical Association [15]. Subsequently, the participants randomly were divided into two groups where Group A (n=15) received Pilates exercise therapy, while Group B (n=15) underwent gym ball exercises. Both groups received the specified interventions for a total of 24 weeks. The assessment of both groups' outcomes involved evaluating the degree of discomfort using the Moos Menstrual Distress Questionnaire (MDQ) and measuring pain levels through the Visual Analog Score (VAS) at the baseline and also after 12 weeks of intervention. The materials used in the study included Swiss balls and exercise mats.

Procedure

Group A underwent a regimen of Pilates exercises which consisted of Pilates pelvic bridge, Single leg stretch, Double leg stretch, and Chest lift 15-minute session per day, three days a week, for a total of 12 weeks. Exercise interventions for primary dysmenorrhea are carried out for a period ranging from four to 12 weeks with more noticeable changes appearing in 12 weeks duration (three sessions per week) according to previous research. It is also suggested that even a 1 cm change in the VAS scale is considered clinically significant [16]. In Group A, each exercise involved a 10-second hold repeated 10 times per day. On the other hand, Group B followed a program of gym ball exercises consisting of Back Extension, Hamstring curls, Wall Squats, and Plank using Gym balls for 15 minutes per session, three days a week, over 12 weeks. Each exercise consisted of a 10-second hold repeated 12 times per set, with a total of three sets per day. Written informed consent was secured from the subjects for using their images while performing the exercise intervention in both groups which can be utilized when the research is submitted for publication. The following exercises were performed by Group A subjects.

Outcome measures

The outcome measure used in the study includes Moos MDQ for assessing the degree of discomfort and VAS for evaluating the intensity of pain.

Moos MDQ was developed by Moos in 1968 and 1977. It consists of 47 items aimed at evaluating menstrual cycle symptoms, both in a cross-sectional and longitudinal manner. Through factor analysis of these symptoms, eight distinct scales have been identified: Pain, Concentration, Behavior Change, Autonomic reactions, Water retention, Negative affect, Arousal, and Control [17].

VAS was used to assess the intensity of menstrual pain. During the menstrual phase, women were asked to assess the intensity of their current menstrual pain using a 10-point VAS ranging from “No Pain” to “The most severe pain I have ever felt.” None of the women reported using pain-relieving medication on the day of evaluation [18].

The data that was gathered was organized and examined using a combination of descriptive and inferential statistical methods. All the variables were evaluated utilizing the Statistical Package for Social Science (SPSS) version 24 (IBM Corp., Armonk, NY). To assess any statistical distinctions within the groups, a paired t-test was utilized. To determine if there were any statistical variations between the groups, an independent t-test, also known as a student t-test, was employed.

## Results

When comparing the average values on the Visual Analog Scale between Groups A and B, it was observed that both groups exhibited a significant reduction in mean values in the post-test. Notably, Group A, which performed Pilates exercises, showed a lower mean value of 2.60 compared to Group B, which performed Swiss Ball exercises and had a mean value of 5.46, with a significance level set at p ≤ 0.001. As a result, the null hypothesis was rejected, showing that Pilates exercises were more effective in reducing scores on the VAS (Table 1).

**Table 1:** Comparison of visual analog scale score between Group A and Group B in pre and post test VAS - Visual Analog Scale *** p ≤ 0.001

VAS	Group A		Group B		t-test	df	P-value
	Mean	S.D	Mean	S.D			
Pre-test	8.13	1.18	7.86	0.833	0.712	28	0.482
Post-test	2.6	0.985	5.46	0.743	-8.99	28	0.000***

Similarly, when comparing the mean values on the Moos MDQ between Groups A and B, both study groups demonstrated a significant reduction in post-test mean values. Specifically, Group A, engaged in Pilates exercises, exhibited a lower mean value of 79.33, while Group B, practicing Swiss Ball exercises, had a higher mean value of 103.26, with a significance level set at p ≤ 0.001 (Table 2). Therefore, the null hypothesis was rejected, suggesting that Pilates exercises were more effective in reducing scores on the Moos MDQ.

**Table 2:** Comparison of Moos menstrual distress questionnaire score between Group A and Group B in pre and post test Moos MDQ - MOOS Menstrual Distress Questionnaire *** p ≤ 0.001

Moos MDQ	Group A		Group B		t-test	df	P-value
	Mean	S.D	Mean	S.D			
Pre-test	107.2	20.86	107.6	18.39	-0.056	28	0.956
Post-test	79.33	15.1	103.26	17.62	-3.99	28	0.000***

Furthermore, when comparing the pre and post-test results within both Group A and Group B for both the VAS and Moos MDQ scores, a high difference of significance in mean values was observed at p ≤ 0.001 (Tables 3, 4).

**Table 3:** Comparison of visual analog scale score within Group A and Group B in pre and post test VAS - Visual Analog Scale *** p≤ 0.001

VAS	Pre-test		Post-test		t-test	P-value
	Mean	S.D	Mean	S.D		
Group- A	8.13	1.18	2.6	0.985	28.83	0.000^***^
Group- B	7.86	0.833	5.46	0.743	12.61	0.000^***^

**Table 4:** Comparison of Moos menstrual distress questionnaire score within Group A and Group B in pre and post test Moos MDQ - Moos Menstrual Distress Questionnaire *** p≤ 0.001

MOOS MDQ	Pre-test		Post-test		t - test	P-value
	Mean	S.D	Mean	S.D		
Group- A	107.2	20.86	79.33	15.1	11.67	0.000^***^
Group- B	107.6	18.39	103.26	17.62	5.89	0.000^***^

## Discussion

Primary dysmenorrhea is a significant concern for women and often leads to frequent visits to healthcare professionals. Consequently, it is crucial to explore treatment options that offer comparable effectiveness with fewer side effects, all while being cost-effective. This study included a sample of 30 female participants aged between 17 and 25 years who experienced primary dysmenorrhea. Participants were assigned randomly into either Group A (Pilates exercises) or Group B (gym ball exercises). Both groups underwent their respective exercise regimens for 12 weeks, with Pilates and Gym ball exercises. The study employed two primary outcome measures: the VAS to assess pain intensity and the Moos MDQ to evaluate menstrual distress. These measures were administered before and after the 12-week intervention.

These results align with a study by Alikiani et al. [19], which reported that 10 weeks of Pilates training for primary dysmenorrhea led to a notable increase in immunoglobulin and progesterone hormone levels. This suggests that these exercises may contribute to a decrease in renin levels and an increase in estrogen and progesterone [20,21]. Furthermore, our study's outcomes are in line with the findings of Izzo and Labriola, who proposed that the enhanced blood flow and metabolism at the pelvic level during Pilates exercises could positively influence dysmenorrhea [22].

Moreover, the research conducted by da Fonseca et al. provided further evidence supporting the benefits of Pilates exercises. Their findings indicated that Pilates exercises not only enhanced muscular flexibility but also reduced the pain and other issues occurring in dysmenorrhea. Additionally, these exercises had a positive impact on the overall quality of life, establishing Pilates as a viable substitute for managing the symptoms of primary dysmenorrhea [23].

In accordance with the fundamental principles of Pilates, including centering, precision, concentration, and controlled breathing, this approach has a holistic impact on both physical and psychological well-being. It has been observed to lead to highly significant alterations in concentration, a reduction in negative emotional effects, and positive behavioral changes [24]. One plausible explanation for these effects is the phenomenon of exercise-induced analgesia. Through Pilates, patients may experience an increase in their pain threshold as a result of adjustments in their endogenous pain control mechanisms. This adjustment could lead to the release of neurotransmitters like norepinephrine, serotonin, enkephalins, and dopamine, which acts to slow down and regulate pain perception [25,26].

On the other hand, the mechanism underlying the effectiveness of gym ball exercises lies in their ability to enhance balance, stability, and blood circulation in the abdominal, pelvic, and lower limb regions, thereby alleviating menstrual pain. In the case of Pilates, it is effective in managing primary dysmenorrhea by directing blood flow to pelvic organs, facilitating the removal of waste products and blood clots that may have formed during menstruation.

The outcomes of the current study revealed a significant improvement with both pain levels and overall quality of life in Group A, which received Pilates treatment, with a significance level of P < 0.001 after 12 weeks of intervention. In comparison, Group B, which was treated with gym ball exercises, showed a minimal improvement in levels of pain and quality of life relative to Group A. The findings of this empirical study provide valuable insights into the management of primary dysmenorrhea through exercise therapy. Notably, both Pilates exercises and gym ball exercises were beneficial to reduce pain intensity and menstrual distress, as indicated by the significant decreases in post-test scores within each group.

However, the key highlight of this study is the comparison between the two exercise modalities. Pilates exercises emerged as the more effective option in managing primary dysmenorrhea, as evidenced by the lower post-test mean values on both the VAS and Moos MDQ when compared to gym ball exercises. This outcome suggests that the specific movements and principles inherent to Pilates, such as core strengthening and flexibility, may have a superior impact on relieving the manifestations of primary dysmenorrhea.

The observed effectiveness of Pilates exercises could be attributed to their ability to enhance core stability and improve overall muscle strength and flexibility, which may aid in reducing pelvic pain and discomfort associated with menstruation. Additionally, the mind-body connection fostered by Pilates may contribute to pain perception modulation, leading to better pain management.

Nevertheless, it is essential to acknowledge some limitations of this study, such as the relatively small sample size and the lack of a control group. Future research should aim to replicate these findings with a larger and more diverse participant pool, including a control group receiving no exercise intervention.

This empirical study highlights the potential benefits of exercise therapy, in the management of primary dysmenorrhea. The results suggest that incorporating Pilates into a regular routine may be a valuable non-pharmacological approach for women seeking relief from menstrual cramps. Further investigations and long-term follow-up studies are warranted to confirm and expand upon these promising findings.

## Conclusions

In conclusion, this empirical study comparing pilates and gym ball exercises as interventions for primary dysmenorrhea has shed light on effective non-pharmacological approaches to managing this common and painful condition. The findings provide valuable insights into the potential benefits of exercise therapy for individuals dealing with menstrual cramps. However, it's essential to recognize the need for further research with larger and more diverse participant populations and control groups to validate and build upon these findings.
